# Guidance for producing a Campbell evidence and gap map

**DOI:** 10.1002/cl2.1125

**Published:** 2020-11-19

**Authors:** Howard White, Bianca Albers, Marie Gaarder, Hege Kornør, Julia Littell, Zack Marshall, Christine Mathew, Terri Pigott, Birte Snilstveit, Hugh Waddington, Vivian Welch

**Affiliations:** ^1^ Campbell Collaboration New Delhi India; ^2^ Centre for Evidence and Implementation London U.K; ^3^ International Initiative for Impact Evaluation New Delhi India; ^4^ Norwegian Institute of Public Health Oslo Norway; ^5^ Bryn Mawr College Bryn Mawr PA USA; ^6^ McGill University Montreal Quebec Canada; ^7^ Bruyère Research Institute Ottawa Canada; ^8^ Georgia State University Atlanta GA USA; ^9^ International Initiative for Impact Evaluation London United Kingdom; ^10^ London International Development Centre London United Kingdom; ^11^ Campbell Collaboration Ottawa Canada

## Abstract

Evidence and Gap Maps (EGMs) are a systematic evidence synthesis product which display the available evidence relevant to a specific research question. EGMs are produced following the same principles as a systematic reviews, that is: specify a PICOS, a comprehensive search, screening against explicit inclusion and exclusion criteria, and systematic coding, analysis and reporting. This paper provides guidance on producing EGMs for publication in Campbell Systematic Reviews.

## 1 METHODS BRIEF: CAMPBELL EVIDENCE AND GAP MAP (EGM) GUIDANCE

### 1.1 Ten tips for producing EGMs

#### 1.1.1 About EGMs

EGMs are a systematic evidence synthesis product which display the available evidence relevant to a specific research question. The scope of a map is generally broader than that of a systematic review.

EGMs are used to identify gaps requiring filling with new evidence, collections of studies for review and increase the discoverability and use of studies by decision‐makers, research commissioners and researchers. They also highlight reviews which can be used to generate higher‐level evidence products such as guidelines.

This methods brief contains 10 tips for producing a Campbell EGM. For more details read the full Campbell EGM Guidance document.

**Ten tips**

1. *Get the framework right*. Determining the map framework is the most important stage of map development and best done through a consultative process with key stakeholders, especially the commissioner of the map. It is important to pilot the proposed framework. Piloting is an iterative process which may take several rounds, which can be characterised as “revise, refine and define”.
2. *Fully comprehensive and mutually exclusive categories*. The set of interventions and of outcomes should fully cover the desired scope of the map (fully comprehensive) but not overlap with one another (mutually exclusive). A single intervention or outcome should clearly fit into a single subcategory without ambiguity as to its proper home. The implication is that many single studies will be coded for just one intervention. Avoid over‐coding: that is putting a single intervention into multiple intervention categories. Multicomponent interventions and systematic reviews are likely to be coded for several interventions. For filters it is useful to add “not reported/not clear” as a category for items such as race or sex.
3. *Produce a dictionary of terms*. A dictionary of all terms used in map—that is intervention and outcome labels as well as filters—is useful for both coders and users. Good practice is for the dictionary to give an example of each label from a study in the map. Locating such studies also contributes to the data cleaning process.
4. *A few big holes rather than many small holes*. The target for the framework is 4–6 row and column heads each with 4–6 subcategories. If subcategories are not used the target is 12–15 row and column heads. Having more headings that this makes the map difficult to navigate for users. Moreover, there is more likely to be ambiguity or missing categories with a large number of very specific headings rather than a smaller number of broader headings. During piloting if items are found which do not fit into the existing framework, consider broadening definitions (widening the holes) rather than adding new subcategories.
5. *The importance of stakeholder consultation*. Stakeholder consultation is important in determining the scope of the map, developing the framework, and interpreting the findings. But there are drawbacks. For example, stakeholder consultation will often create pressure for more categories as they want to see “their interventions” named. But this pressure needs to be weighed against the disadvantages of a cumbersome framework.
6. *Be realistic on the timeline*. Although the coding, analysis and report writing are easier for a map than a review, the broader scope means there are many more studies to screen and code. So, the overall time required may not be much different to that for a full review.
7. *Produce the map in stages*. It can be a good idea to produce the map in stages. A first stage can map studies using a “low hanging fruit approach”, for example, relevant Campbell reviews and the eligible included studies in those reviews, or eligible studies in a single database such as the 3ie evidence hub. Have a staged approach allows an intermediate output, and for some user testing with an actual map rather than the proposed framework which seems a bit abstract to intended users.
8. *Consider different visual representations of the map*. Most maps have primary dimensions, which are interventions and outcomes for effectiveness maps. However, it can be useful to present the map with other row and column headings, for example, interventions in rows and regions as headings, as a way of seeing the geographical distribution of evidence.
9. *Add a bit of colour to the report*. The report of the map findings is a descriptive analysis of the distribution of studies. The report can be both more interesting and useful if some depth and colour is added, which can be as simple as a box listing study titles for certain categories. If it is a field in which certain study designs are rare, then a box or the text may elaborate on a study using that design to illustrate its use. If a common problem is identified in the critical appraisal of included studies some examples can be provided of this problem and studies which overcome it.
10. *Innovative reporting*. The report can also be made more interesting by more innovative analysis. There are approaches used by bibliometricians which could be more widely used in reporting maps, for example, network analysis.

## 2 SUMMARY

EGMs are a systematic evidence synthesis product which display the available evidence relevant to a specific research question.

The scope of a map is generally broader than that of a systematic review. We distinguish between EGMs which contain primary studies and reviews, mega‐maps which include reviews and other maps, and maps of maps which contain only other maps.

EGMs are used to identify gaps requiring filling with new evidence, collections of studies for review, and increase the discoverability and use of studies by decision‐makers, research commissioners and researchers. They also highlight reviews which can be used to generate higher‐level evidence products such as guidelines.

Maps are typically shown as a matrix. The most common map is a map of effectiveness studies (or an “effectiveness map”, although it does not show effectiveness), although maps may be made of any body of literature. An effectiveness map is most commonly drawn with interventions as row headings and outcomes as column headings, both may be subdivided into subcategories. The cells contain the studies relevant to that intervention/outcome combination. The map is interactive so users can click on the cell to access the studies.

There are also filters, such as study design, country or region, and subpopulations, so users can see a subset of studies meeting certain criteria.

The row and heading titles and the filters are referred to as the map framework. Determining the map framework is the most important stage of map development and best done through a consultative process with key stakeholders. Stakeholders may also be involved in other stages of the process, especially use.

The search, screening and coding follow the usual systematic review approach. Critical appraisal can also be included and shown in the map.

The map is accompanied by a descriptive report providing an overview of the studies, for example, frequency plots.

Producing a map usually requires of team of one or two principal investigators (PIs; content expertise, and knowledge of evidence synthesis) and a team of two junior researchers or research assistants. A map may be expected to take 6–12 months to complete.

## 3 INTRODUCTION AND OVERVIEW

An EGM is a systematic presentation of all relevant evidence of a specified kind for a particular sector, subsector or geography. Examples of maps include “Evidence and gap map of studies assessing the effectiveness of interventions for people with disabilities in low‐and middle‐income countries” (Saran et al., 2020) and “The impacts of agroforestry on agricultural productivity, ecosystem services, and human well‐being in low‐and middle‐income countries: An evidence and gap map” (Miller et al., 2020).

Produced using the same systematic approach as a systematic review, EGMs usually show what evidence is there, not what the evidence says. Following systematic processes takes time, so while a map might be produced in 3 months, 6–12 months is generally a more realistic timeline.

The scope of an EGM is typically much broader than that of a systematic review. A map typically contains systematic reviews and primary studies, but may include only one of these, and may sometimes also include other maps.

Campbell EGMs include a visual presentation of the evidence as a matrix. Usually the matrix shows intervention categories as rows and outcomes as columns.^1^

**Figure 1:**
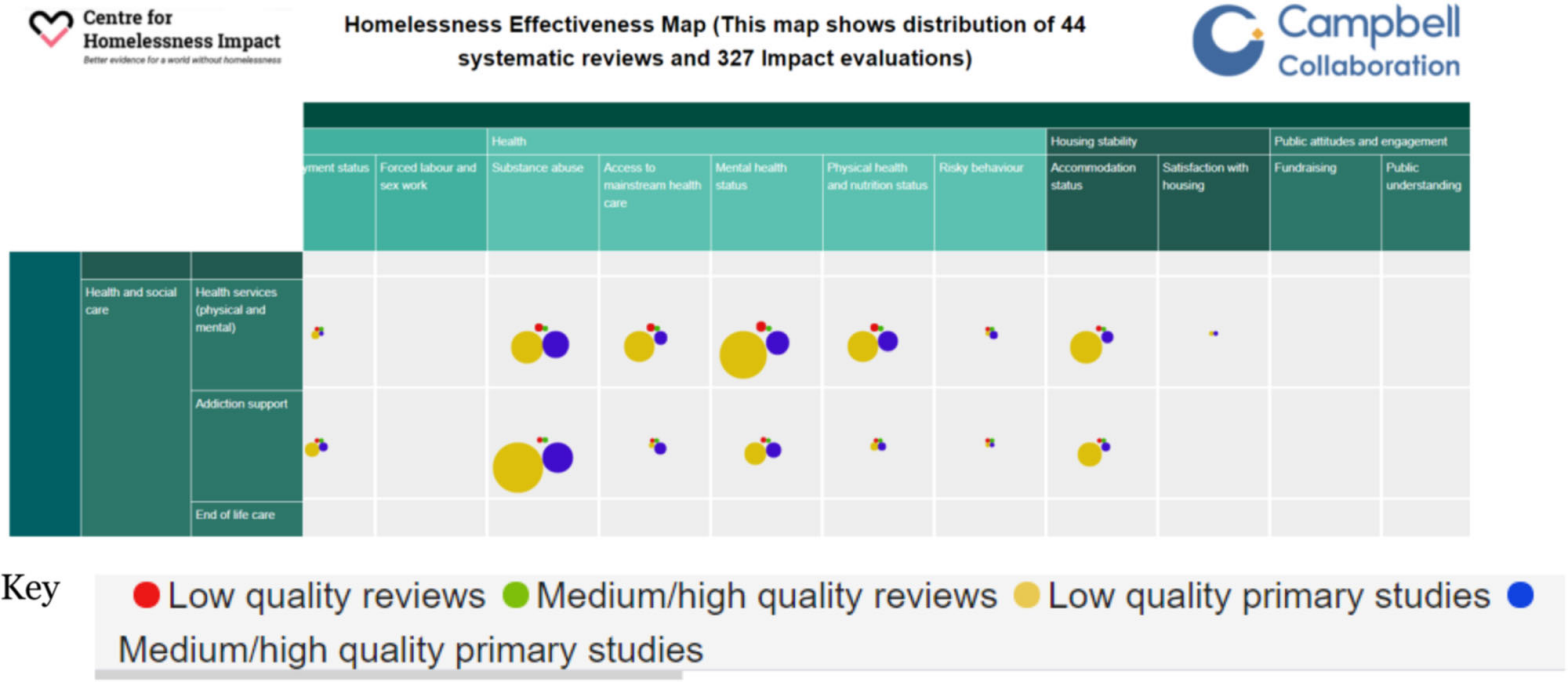
Screenshot of a section of homelessness evidence and gap map

Figure 1 shows an example of a section of a map on homelessness. The section shown illustrates an intervention category (health and social care) and its first three subcategories (health services, addiction support and end‐of‐life care), as well as three of the outcome domains (health, housing stability, and public attitudes and engagement) and their corresponding outcome subdomains. The bubbles in each cell show the studies included in the map. The separate bubbles show type of study (primary study and systematic review) and confidence in study findings based on critical appraisal of each study. For example, in Figure 1, the red bubble corresponds to systematic reviews for which we have low confidence in study findings. The larger the bubble the more studies there are.

While the intervention‐outcome matrix is the most common representation of an EGM, different representations can also be useful—for example, showing availability of studies for interventions against global region.

The map may have additional dimensions capturing study or intervention characteristics, such as study design, location and population subgroup which can be applied as filters so as to only show evidence for that filter (e.g., randomized controlled trials [RCTs] or Asia). The map is interactive so that users may click on entries to see a list of studies, and on study names to access the study or a database record for the study.

Just like a systematic review, an EGM can be produced to address any research question. The type of evidence to be included in the map is determined according to the research question. Maps may be produced, for example, of studies of effects, prevalence, implementation or barriers or facilitators. The title of the map should make clear the type of evidence being included. Some research questions may require more scoping to develop the map framework and inclusion criteria.

There are many approaches to evidence mapping (Saran & White, 2018). This guidance presents advice on preparing EGMs to Campbell standards (Campbell Collaboration, 2019). Research teams constructing a Campbell map should also consult the Campbell Collaboration Checklists for the Production and Report of Reviews (White et al., 2018a, 2018b).

## 4 WHY PRODUCE AN EGM?

Mapping is standard part of the systematic review process, and most reviews will conduct some sort of mapping even if they do not publish a map. Many organisations have produced maps of different types. A review of these may be found in Saran and White (Saran and White, 2018).

Maps can be used for various purposes, and the main purpose may vary by type of map. The main uses of EGMs:

- Guide users to available relevant evidence to inform intervention and design and implementation. Very often users (both researchers and decision‐makers) are unaware of the extent of the evidence base, so maps are a way of increasing the discoverability, and hence use, of that evidence. Country evaluation maps, such as that for Uganda, make available over 500 evaluations many of which were little used as they were not know and not readily discoverable.
- Identify existing high‐quality reviews as a basis for evidence summaries for policy purposes or to populate evidence portals (e.g., the UK Centre for Homelessness Impact's Intervention tool).
- Tell implementing agencies where there is no relevant evidence for their interventions. Agencies committed to evidence‐based interventions should be aware when they are operating in an “evidence free” area (or one with only weak evidence) so they can act accordingly (i.e., shift to a different, evidence‐based intervention, or collect evidence for the intervention they are supporting). The effective altruism charity, Giving Evidence, uses maps for this purpose with its clients.
- Identify research gaps for new primary research and new synthesis. This enables a strategic, policy‐oriented approach for research commissioners to decide what research to commission and researchers what research to do. A coordinated research programme would begin with evidence mapping, identify gaps for new primary research, and conduct synthesis on the basis of the primary studies included in the map. The UK Youth Endowment Fund has commissioned a map of interventions to prevent youth crime for this purpose. UNICEF commissioned the map of violence against children based on the results of the child welfare mega‐map, which they also supported.

EGMs most commonly have a primary purpose to allow a more strategic approach to the commissioning research by identifying areas for which there are no or few primary studies, or many studies but no systematic reviews. They may also identify areas in which there are many reviews and so a review of reviews may be appropriate. Research commissioners use this information, along with their own knowledge priorities, to decide what studies to commission.

While informing research is a primary purpose of EGMs, they can also be used for strategy and programme development by identifying relevant evidence. EGMs save strategy and programme developers the time and effort of screening studies from search engines such as Google Scholar, and more effectively and efficiently guide them to relevant evidence for the interventions and outcomes of interest than their own efforts to find these studies.

Where maps are being used as a basis for identifying reviews to be undertaken, maps can improve the efficiency of review production in two ways. First, the included studies for a review are the relevant studies identified in a map.^2^ Where the coding for maps is available for the review team then the workload for coding is reduced.

It is particularly useful to identify areas in which there is no evidence. Agencies cannot claim to be doing evidence‐based programmes in such areas, and should be using any new or existing programmes to collect evidence of effectiveness.

## 5 TYPOLOGY AND TERMINOLOGY

The studies included in a map may vary according to its scope (i.e., the breadth of sectoral and geographical coverage). The following three names are used by Campbell:

- *Evidence and gap map*: A map of a specific sector or subsector which typically includes both systematic reviews and primary studies. For example, “The impacts of agroforestry on agricultural productivity, ecosystem services, and human well‐being in low‐ and middle‐income countries: An evidence and gap map” (Miller et al. 2020) and “Interventions for adults exposed to war and armed conflict: An evidence and gap map” (Farina and Maynard, 2019).
- *Mega‐map*: A map with broader scope covering a large sector or several sectors that includes only systematic reviews and other maps. For example, “Mega‐map of systematic reviews and evidence and gap maps on the effectiveness of interventions to improve child welfare in low‐ and middle‐income countries” (Saran et al., 2019).
- *Map of maps*: A map with a very broad scope covering many sectors that only includes other maps. For example, “A map of evidence maps relating to low‐ and middle‐income countries” (Phillips et al., 2017).

The name may also be varied to give a clearer indication of the evidence contained in the map. For example, “The country evaluation map of development interventions in Uganda” or “Uganda evaluation map” is a map of all sorts of evaluation (not just effectiveness studies). Or “The prevalence of violence against children: an evidence map of prevalence studies”.

**Figure 2:**
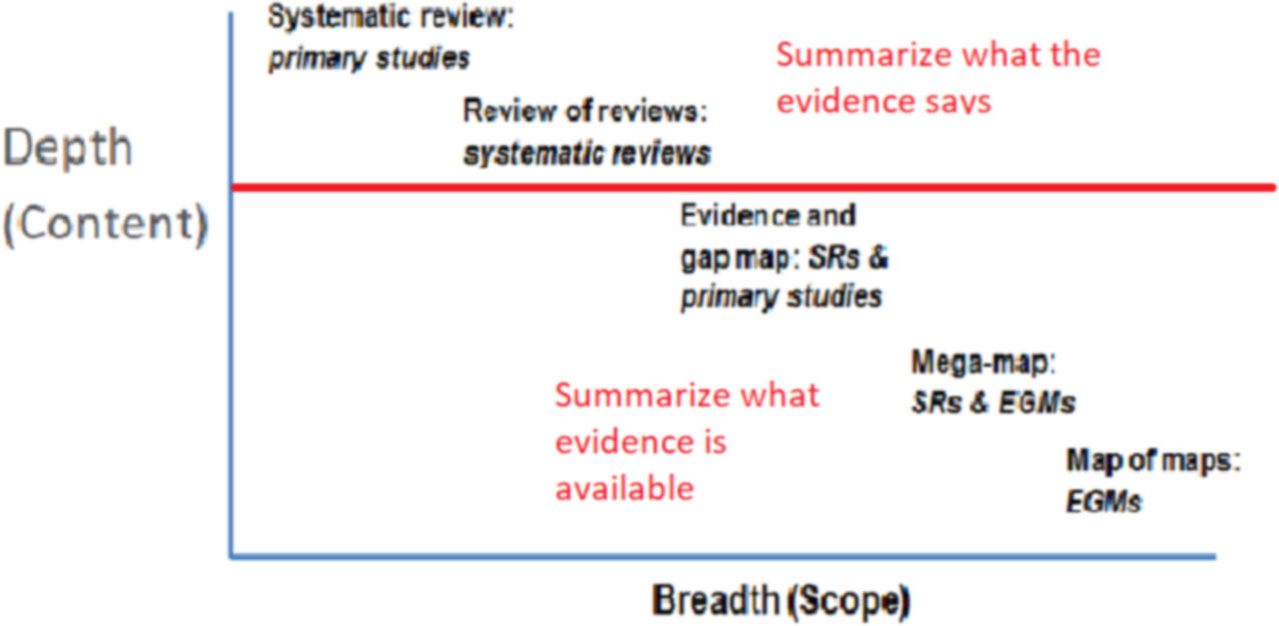
Types of evidence synthesis.Source: Saran and White (2018)

An EGM is one type of synthesis product. There are other types. There is a trade‐off between the depth and breadth of these different types of evidence synthesis product. As shown in Figure 2, the broader the scope (horizontal axis) then the less deep is the analysis (content) of the included studies.

A key distinction is the step between reviews of reviews and EGMs, as the former summarise what the evidence says, whereas the latter show what evidence there is. While reviews may vary in the extent to which they conduct synthesis—some systematic reviews simply summarise each study, so are more akin to an annotated bibliography than a synthesis—they do attempt to summarise what the body of evidence says, which Campbell maps do not.

In addition to the naming conventions presented above, the following terminology is used for Campbell maps:

- *The framework*: The framework for an EGM defines the dimensions of the map: row and column headings and filters.
- *Dimensions*: The two primary dimensions of an EGM are the row and column headings. For an effectiveness map these are: (a) intervention categories, and (b) outcome domains. As shown in Figure 1, intervention categories are usually divided into subcategories, and outcome domains into subdomains. Secondary dimensions are used as filters (see next item).
- *Labels*: The label is the name given to a category or subcategory in the map (e.g., “End of life care” in Figure 1).
- *Filters*: Filters are study characteristics (e.g., study design, country or region, or subpopulation) which may be applied to the map to show only evidence relevant to those filters.
- *Aggregate map*: A map showing the data by category level for rows and columns, rather than subcategory level.

## 6 DEVELOPING THE FRAMEWORK

Developing the framework, in particular the primary dimensions, that is, row and column headings, is the most important, and often the most difficult, part of developing an evidence map.

The framework and PICOS are closely related.^3^ In an effectiveness map such as Figure 1 the rows and columns are the I and the O in the PICOS. More generally, the framework is the basis for the search strategy and coding forms, and for presenting and analysing the findings.

The framework needs to be codable and resonate with users. So, category headings should be one which are readily understandable to those coding the map, and enable users to find studies of specific interventions, or for particular outcomes, where they expect to find them. There is a trade‐off between avoiding jargon but using language familiar to sector stakeholders although it may not be readily understandable by lay users, noting also that that this terminology may vary by country. For example, “contingency management” was the dominant approach to tackling homelessness in the USA for some years. It is not apparent to lay readers what this label means, but will be so to any user working in the sector.

It is also important to avoid overlong labels for the simple reason that they are unlikely to be readable in the visual representation of the map.

In general, the framework should be developed with these principles:

- Where there is an existing, widely accepted international typology for either interventions, outcomes or another primary dimension then this typology should be adopted. For example, the disability EGM (Saran et al., 2020) adopted WHO's Community‐Based Rehabilitation Matrix for both interventions and outcomes.
- However, the research team should assess the suitability of the typology, and revise it as necessary. For example, the EGM of interventions to reduce violence against children (Pundir et al., 2020) drew upon the widely adopted INSPIRE framework,^4^ but modified it to create suitable coding categories.
- Where there is no such international typology, the research team should consult the strategy documents of the main international actors, usually multilateral agencies and global programmes, but also the strategy documents of the agency or agencies funding the map, if any.
- If strategy documents are not available, then it may be necessary to consult project documents of intervention within the scope of the map. A sample should be taken of major funders in the area, including those funding the map.
- Alternatively, a framework can be adopted and adapted from other research studies (maps or reviews). The initial framework used for the map of interventions to improve the welfare of those experiencing or at risk of homelessness was taken from the review by Munthe‐Kaas et al. (2018), though subsequently substantively revised on the basis of expert review and stakeholder consultation.
- If no suitable framework is available then the research team can develop their own by drawing on the range of resources listed above.

In all cases, the framework will be subject to modification through stakeholder consultation, the piloting process and the requirements of the commissioner. Annex 1 provides guidance on how to structure stakeholder consultation.

Stakeholder consultation is an important stage in developing the map, in particular consultation with the funder(s). The advisory group for the map should include a broad range of stakeholders (policy, practitioners and researchers). Advising on the framework is one of their key contributions. Stakeholders will expect to recognise the categories used, and in particular to see “their intervention”.

However, the inputs from stakeholder consultation need to be balanced against the principle of having a manageable and easily navigable map. Sector stakeholders may well push for proliferation to have categories naming specific interventions. The consultation should bring the stakeholders on board with the need to have broader categories which clearly accommodate their interventions of interest. In general, a map should have four to six row headings (categories) and four to six column headings (categories), with each having up to five subcategories. Ideally, a map is 20–25 × 20–25 rows and columns. If subcategories are not used then there can 12–15 categories.

If the numbers are sufficiently small to fit on one screen without scrolling that is an advantage, but generally only be possible for the aggregate map. The map may also be printed, but the print will appear small unless there are few rows and columns.

**Figure 3:**
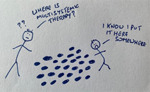
The framework categories should use clear labels and should not be too disaggregated

The general principle is “a few large holes” rather than “lots of small holes”. Too detailed a disaggregation of either of the primary dimension makes the map difficult to navigate and coding more challenging (Figure 3), with greater knowledge and judgement required on the part of the coders.

## 7 STAKEHOLDER CONSULTATION

Stakeholder consultation is an important part of the map, and its role appears at several places in this guidance. This consultation includes defining scope and framework development, engagement in piloting, identify sources for the search such as organisational websites, and discussing and promoting use of the map.

## 8 EGM TITLE AND SCOPE

The title of the EGM should define the scope of the map. It is reported in each of the title registration form (TRF), the protocol and the report.

EGMs have a broader scope than most systematic reviews. Hence the title for an EGM will be broader than that for a review. Mega‐maps and maps of maps have a still broader scope.

The long title provides further information such as the type of studies being included. For example:

- An EGM of studies of the effectiveness of interventions to reduce abuse and neglect of children at risk and vulnerable children (aged 0–11) in high‐income countries.
- An EGM of studies of the prevalence of violence against women and girls in South Asia.
- An EGM of studies of interventions promoting safe water, sanitation, and hygiene for households, communities, schools and health facilities in low‐ and middle‐income countries (L&MICs; Waddington et al., 2018).
- Understanding pathways between agriculture and nutrition: an EGM of tools, metrics and methods developed and applied in the last 10 years (Sparling et al., 2019).

The short title may be centred on population, outcomes or intervention. For example:

- Population‐oriented title: Disability EGM
- Outcome‐oriented title: Child welfare mega‐map
- Intervention‐oriented title: Access to justice EGM

The scope of the map should be determined with the commissioner (if any) and the intended users of the map. The consultation process should be outlined briefly in either or both the TRF and the protocol.

A search should be made for existing and ongoing maps to avoid duplication. It is acceptable that there may be some overlap between maps.

## 9 EGM PICOS

For EGMs of effectiveness studies, the scope is fully defined by the PICOS, which is reported in the TRF, Protocol and Report. Variations on PICOS may be used for maps assessing the evidence base for other research questions. For example, in a map of process evaluations examining implementation issues, then the columns may be barriers and facilitators rather than outcomes. A map of risk factors could have population groups in rows and risk factors in rows, so neither interventions or outcomes appear.

### 9.1 P: Population

Specify the types of populations to be included and excluded. The description of the included population can be broad, for example, “low and middle‐income countries” or “children under 12”. As may also be the case for reviews, the population may be implied by the intervention, for example, “interventions to increase school attendance and enrolment”.

Population subgroups (e.g., women, children, ethnic minorities, conflict‐affected populations and people with disabilities) are usually an important filter. User consultation is helpful in identifying relevant subgroups.

Population may also sometimes identify exclusion criteria if appropriate. For example, an evidence map for homelessness may exclude those made homeless by natural disasters. Maps of education interventions may exclude children with learning disabilities.^5^

### 9.2 I: Interventions

Interventions are usually one of the two primary dimensions of the evidence map, being used as the row headings. An evidence map should preferably have four to five categories each with four to five subcategories, giving around 20 rows. Annex 2 gives the example of a framework for a map on interventions to increase the welfare of those experiencing or at risk of homelessness (White at al. 2020).^6^

As discussed above, the intervention categories are identified by (a) existing frameworks; (b) examination of strategy and project documents, especially those of the commissioner where appropriate; (c) consultation with the commissioner and other stakeholders; and (d) piloting of the initial framework.

Interventions should be clearly defined. They can include policies, programmes and practice. The categories should be fully inclusive of the scope and mutually exclusive—any one intervention should ideally not fall in multiple categories, though of course may do if it has multiple components which is common.

Developing a typology of interventions is possibly one of the important contributions of the map. To assist coders and users it is helpful to develop a dictionary defining the intervention labels. As the map is produced it is also useful to include examples from the map of a study coded under each label. This exercise can help sharpen the definitions, and also be a stage in data cleaning.

### 9.3 C: Comparison

In the case of an effectiveness map then comparison group (e.g., active versus passive) may be used as a filter. Studies which include both active and passive comparison groups (which systematic reviews often will) should be coded under all categories that apply. In the case of an active comparison (i.e., the comparison group also gets an intervention) then the comparison intervention is also coded for that study in the map.

### 9.4 O: Outcomes

Outcomes are one of the two primary dimensions of an evidence map of effectiveness, being used as the column headings. Other examples of column headings are barriers and facilitators in a map of process evaluations and implementation studies (White, Wood et al, 2018) and tools, methods and metrics in a “methods map”.

An evidence map should preferably have 8–12 outcome domains, or 4–6 outcome domains each with 4–6 subdomains, giving around 20–25 columns (see Figure 1 and Annex 2).

As discussed above, the outcome categories are identified by (a) existing frameworks; (b) examination of strategy and project documents, especially those of the commissioner where appropriate; (c) consultation with the commissioner and other stakeholders; and (d) piloting of the initial framework.

### 9.5 S: Study designs

EGMs may be presented for any type of evidence, for example, effects, prevalence, and implementation studies. A key question related to the scope of the EGM is “what sort of evidence will be presented?”. The long version of the map title should give an indication of the sort of evidence being included in the map.

The TRF should give a broad indication of the type of studies to be included, for example, “systematic reviews and primary studies of effectiveness”. The protocol should spell out in detail eligible and ineligible study designs that are the basis for the inclusion and exclusion criteria to be used in screening. Screening tools may be included as an annex.

## 10 SEARCHING AND SCREENING

The search should be conducted and reported to Campbell standards, as laid out in the Campbell Collaboration Checklist for EGMs (White et al., 2018a, 2018b). Main points are that several databases should be searched using documented search strings, results double screened and the search results reported accompanied by the PRISMA diagramme.^7^

Study teams should consult the Campbell Methods Guide “Searching for studies: a guide to information retrieval for Campbell systematic reviews” (Kugley et al., 2017) for further information.

Reference snowballing and citation tracking are an important part of the systematic search strategy. However, since maps usually have hundreds of included studies, there are limits to the feasibility of these approaches. Nonetheless, ensuring that all eligible included studies in the included reviews are in the map (a process referred to as unzipping the reviews) is an important part of the search and screening for map, and often one of the richest sources of primary studies in a well‐reviewed area. It is also recommended to snowball references for the twenty or so most recent studies in the map. Citation tracking is recommended for 10–20 highly cited studies in the map.

Since a primary purpose of maps is as a basis for a consultation to identify research priorities it is important to include on‐going studies. This is done by searching libraries of reviews (e.g., Campbell, Cochrane, EPPI‐centre, Centre for Environmental Evidence, 3ie systematic review repository for reviews of studies conducted in L&MICs) and registries (e.g., Prospero for systematic reviews, American Economic Association RCT Registry, OpenTrials, the Registry for International Development Impact Evaluations for primary studies in International Development, WHO ICTRP).

Just as for a review, the search strategy should be piloted. An iterative process is usually needed to develop the search strategy.

Campbell maintains a list of useful resources organised by sector including databases which are recommended for searching. See: www.campbellcollaboration.org/links

Since the scope of EGMs is usually quite broad, then it is likely that there will be more studies to screen than for a review. A review may typically screen some 2,000–5,000 studies, whereas screening for a map may be 15,000–30,000 studies or sometime more. When there are so many studies to be screened consideration may be given to a machine‐learning assisted search. Even then screening can take 4–6 weeks for full time work, and so in practice likely longer actual time.

## 11 CODING

EGMs require less coding per study than systematic reviews. But a map usually includes more studies than a review, so the workload for coding can be similar.

The coding form should include:

- Basic study characteristics, that is, bibliographic details.
- Primary EGM dimensions (e.g., for an effectiveness map, intervention categories and subcategories, and outcome domains and subdomains). A single included study may span more than one category or domain, and this is to be expected in the case of included reviews and maps.
- Secondary EGM dimensions to be included as filters.
- Data required for critical appraisal if included (see below).

It is generally not recommended to code direction of effect or effect size since Campbell maps do not portray this information. However, this may possibly be done in the case of a mega‐map which includes only systematic reviews. In addition, the evidence that may be prepared for each included study will give information on study contents.

The coding form should be piloted with a small number (20–30) of included studies. The piloting exercise should include the PIs and those who will actually do the coding, if different. Piloting of the framework allows a process of “revise, refine and define”. Each round of piloting may lead to revisions in the framework, refinements of categories, and clarifying definitions of each label. Refinement is usually best a broadening of categories to accommodate interventions not previously identified. It is not good practice to simply add new labels for previously undiscussed interventions as this can rapidly lead to category proliferation to make the map unwieldy.

The final version of the coding form should be included in the protocol.

### 11.1 Data cleaning

As with any data set, the coding data will need to be cleaned once coding is completed. This can be done using usual data coding techniques such as:

- Univariate tabulations of all codes to identify uncoded data, out of range codes and investigate unexpected patterns in the data.
- Examine a random sample of coded records, systematically checking in all records any code which appears to incorrectly used by the coders.

It is also useful to ask trusted users to explore the map prior to launch and report any issues they find with the coding.

### 11.2 Screening and coding included systematic reviews and EGMs

Systematic reviews and EGMs may be coded in one of two ways:

- According to their eligibility criteria (PICOS)
- According to their included studies

The main advantage of the former approach is ease as screening and coding can be based of the PICOS of the review or map being screened or coded. However, the disadvantage is that the study may be included in the map but not include any studies meeting the inclusion criteria of the map. A common example is an EGM of evidence related to evidence from L&MICs. Such a map would include any relevant systematic review with a global scope and so in principle contains studies from developing countries but yet *not in fact include* existing primary studies from a L&MIC since none were found. Experience shows that map users are surprised, disappointed or annoyed when finding a review in the map which does not contain any primary studies that are relevant (i.e., which meet the map's inclusion criteria).

Hence the second approach—that is coding against included studies—may be better. This means that the included studies in the systematic review need to retrieved and coded to determine the coding of the systematic review. Of course, these primary studies need to be coded anyway as they will also be in the map, unless they are excluded on grounds of study design or, date of publication, or other grounds.^8^

This approach will mean that reviews eligible on their PICOS but not their included studies are not included in the map, which will always be the case for empty reviews (i.e., a systematic review which finds no eligible studies). Another possibility is to show reviews which are eligible on their PICOS but include no studies eligible for the map on the map, but coded with a different colour to reviews which have eligible included studies.

## 12 CRITICAL APPRAISAL OF INCLUDED STUDIES

Critical appraisal of included studies is recommended for Campbell EGMs but not mandatory. It is also possible to conduct critical appraisal of some studies, for example, systematic reviews, while not conducting this for other, for example, primary, studies.

The options are:

- Critical appraisal of all included studies. This is recommended for Campbell EGMs but not mandatory.
- Conducting critical appraisal of systematic reviews but not of primary studies. The main rationale for this approach is workload related. There are many more primary studies so critically appraising them can add considerably to the work burden. If critical appraisal of primary studies is not done, then it recommended that primary study study design is coded and included as a filter.
- Not conducting any critical appraisal, which is not recommended since it is relevant to know not only how much evidence there is and what it covers but also the confidence we can have in findings from that evidence.

Where critical appraisal of any included studies in the map is done then the results can be shown as either in the colour coding scheme for the map or as a filter.

There are many critical appraisal checklists available for both primary studies and reviews. See for example the tools of the Critical Appraisal Skills Programme,^9^ Lewin et al. (2009) for reviews of effects, Lewin et al. (2015) for qualitative evidence syntheses, Shea et al. (2016), and the list provided by the Cardiff University Specialist Unit for Review Evidence.^10^ AMSTAR 2 (Shea et al., 2016) is a commonly used tool for critical appraisal of systematic reviews.

Since an EGM typically includes both reviews and primary studies, and may include multiple study designs (e.g., experimental and nonexperimental) then more than one check list will need to be employed.

The critical appraisal tools should be specified in the protocol. Tool developed or adapted specifically for the map should be included in the annex.

## 13 EGM PROTOCOL

The protocol for an EGM should include:

- A clear indication of the scope of the map by reporting the PICOS, adapted as needed be to the type of research question.
- The framework: in the case of a map of effectiveness studies, this is the intervention and outcomes, as well as any filters. It is preferable to have an annex which includes definitions for all labels. Annex 2 provides a sample framework with definitions, in which examples are also given of a study in the map coded under each label.
- The search strategy.
- The coding form, including any tool used for critical appraisal.
- An analysis and reporting plan.

It is strongly recommended to pilot the search strategy, the screening tool and the coding form before submission of the protocol. The piloting of the first two provides studies to include to illustrate the items (labels) in the coding tool. The exercise of identifying studies for each label is a useful part of the “revise, refine and define” iterative process in finalising the coding tool.

Authors should consult the Campbell EGM conduct standards checklist for a complete list of what to include in the protocol (White et al., 2018a).

## 14 EGM REPORT

The online interactive map is accompanied by a descriptive report to summarise the evidence for stakeholders such as researchers, research commissioners, policy makers and practitioners. Relevant guidelines on conduct and reporting standard for Campbell EGM can be found here (White et al., 2018a, 2018b). Analysis of studies included in the EGM is descriptive and report should capture following information.

- Description of the EGM methodology.
- Main findings in terms of spread and concentration of evidence across intervention and outcome categories highlighting important evidence gaps and trends identified in the research literature.
- Additional findings from filters such as study design, geographical location (ideally both regions and countries), population, confidence in study findings (assessed through standardised checklists), funding and implementing agency for the included studies.
- Implications for policy and future research and key recommendations.
- A Plain Language Summary highlighting key findings in plain language, without use of any jargon.

The total number of studies in a map is often the main findings, possibly broken down as primary studies and systematic reviews. This information may be included in the title of the online map (see Figure 1). It is useful to do that since a study may appear in multiple cells so simply counting bubbles give an inaccurate impression of the amount of evidence available.

The authors need to decide whether to distinguish studies and papers, that is, there may be several papers from a single study. The *Chez Soi* study of a Housing First intervention in five Canadian cities accounts for around 20 primary studies in the map of homelessness. In systematic reviews, it is necessary to identify papers from the same study, especially if meta‐analysis is being performed. This is not strictly necessary in the same way for a map, and is more difficult for a map than a review on account of the large number of studies typically included in a map and the fact that the coding is not as detailed. However, differentiating between papers and studies can be useful for users and is recommended especially if it is planned to use the map to generate reviews.

The EGM report should discuss the distribution of evidence and identify the main gaps in the evidence base. Tabulations and cross tabulations should be presented of the primary and secondary dimensions. The time trend of publication is usually presented.

Care should be taken in how the map is presented. Where the map only shows reviews, not primary studies, then the map shows “gaps in evidence synthesis” not “gaps in evidence”. If a map has both reviews and primary studies, then where a cell contains a review but no primary studies then it is correct to say there is no evidence since these reviews are empty reviews with respect to that intervention/outcome combination (which we can see as there are no primary studies).

Authors are encouraged to explore engaging and innovative ways of presenting the data. In addition to histograms of the distribution of studies presentations may include, for example, a global map showing the distribution of studies, possibly using a heat map approach, and a network diagram of authors or their institutions. Campbell reviews currently use a limited range of bibliometric methods. Evidence maps present an opportunity to borrow more from this closely related field.

## 15 FORMATS

The formats for the TRF are available on the *Campbell Systematic Reviews* website.^11^ The protocol and report formats are embedded in Archie (see next item).

## 16 SOFTWARE

The Archie review management system for the protocol and final report is required to produce Campbell evidence synthesis products. The TRF is submitted as a word file.

A range of options are available to generate maps. EPPI‐reviewer can be used to generate the online map from a JSON file, which can be exported from EPPI‐reviewer. Teams may also apply to 3ie to use their mapping software,^12^ or write their own code using packages such as R or Stata.

EPPI reviewer can be used for search, screening and coding, and the data thus used to generate the online evidence map and to produce the tables and figures for the descriptive report.

There are also increased opportunities for using machine learning to produce and update maps. For example, EPPI Reviewer has machine learning functionality for both searching (using the dataset of Microsoft Academic) and screening. Since machine learning requires training data to start it off the application to updates is clear, and opens up the path for living maps.

There are also automated data extraction tools used for qualitative data analysis such as Atlas‐ti, but the ability of these to successfully carry out the thematic coding needed for, for example, intervention and outcome categories is still under development.

Currently it is recommended to use any machine learning application in conjunction with human search, screening and coding.

## 17 THE ONLINE EVIDENCE MAP

Campbell partnered with EPPI reviewer to produce a user interface, based on EPPI‐reviewer database for mapping. In the example below on interactive map, outcomes were used columns and intervention as rows. The categorisation system for rows and columns (called branches in the EPPI Reviewer coding system) used in EPPI Reviewer as codes can only be one or two level deep and need to be uniform with all subcategories same or with similar layout. The columns and rows are defined in EPPI‐reviewer as “codes” and the circle represent quality and quantity of included studies in each cell.

Coded data is then exported via “coding report” in JSON format from EPPI‐reviewer. The EPPI Centre team responsible for EPPI Reviewer are available to discuss issues in designing the coding structure to achieve the desired visualisation with authors of Campbell EGMs.

**Figure 4:**
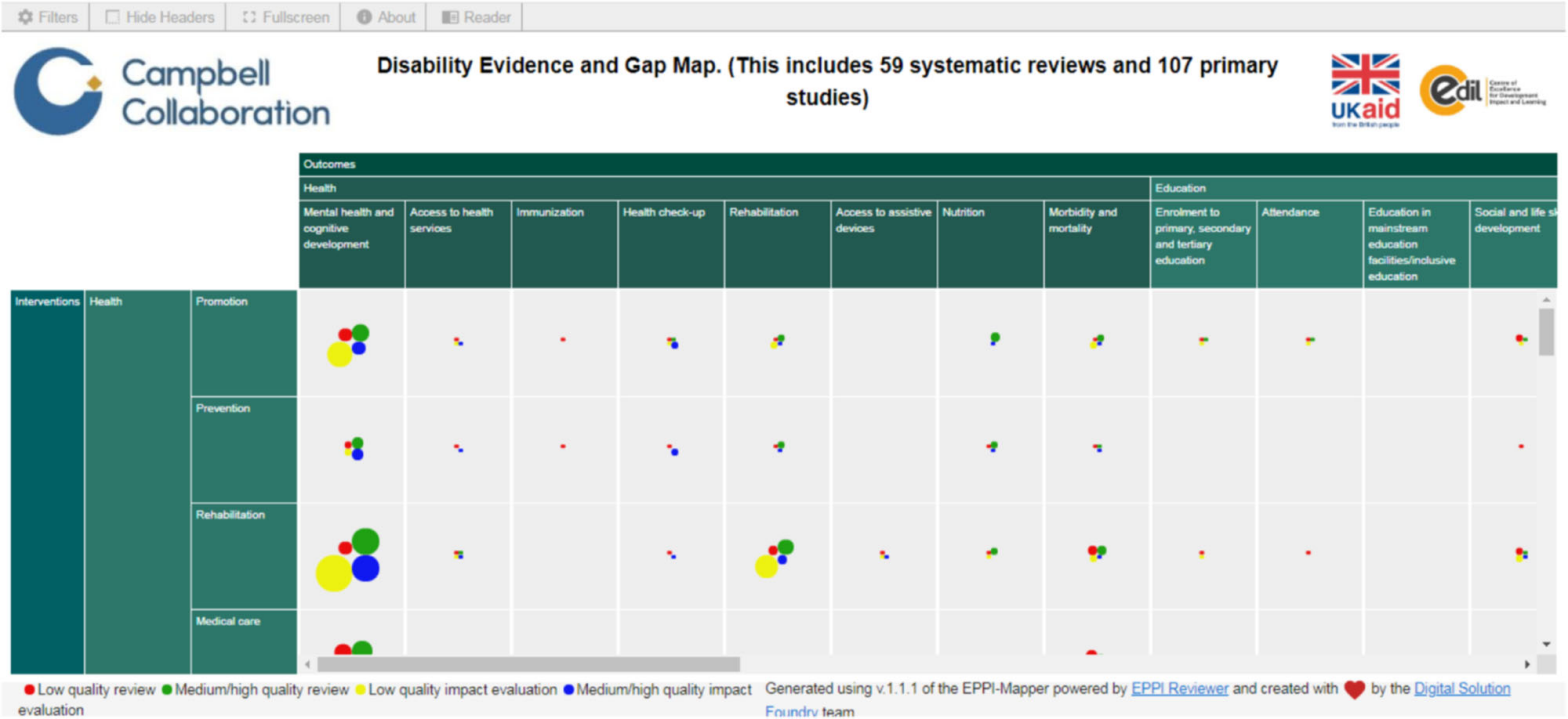
Snapshot of an interactive Campbell evidence and gap map

The exported data is then imported into EPPI‐Mapper (mapping utility) to create an interactive map (see sample map in Figure 4). Map can be customised and published online. By clicking on any cell, a pop‐up window opens displaying the list of studies included in that cell. Maps can be filtered depending on the data behind it and is displayed in four different styles.

Reader function in the online map allows users to search for specific types of studies within a data set, which then displays studies with bibliographic information. Users can also locate studies by clicking on any cell.

It is strongly recommended to generate a test map once the pilot studies are coded to ensure that the desired presentations can be generated. Providing this test map to stakeholders for consultation and feedback may identify issues related to coding interventions, outcomes or filters. Viewing the map can have implications for the content of the coding form and how it is structured in the coding software.

## 18 DATABASE RECORDS

All included studies for studies coded in EPPI Reviewer have a record in the underlying Global Evidence Database.^13^ This record is automatically generated by EPPI Reviewer from the coding once the map is published.

The record contains the information that has been coded. Database entry writers may be different to the team performing screening and coding, as entry writing it is a more specialised skill set.

In addition to that basic information it is highly recommended that the record also contain:

- Description of the intervention
- Context
- Study design
- Main findings

It may often prove practical to complete the basic coding to complete and publish the map, and then produce the additional content for the database records.

Planning time and budget for EGMs should allow for this phase.

## 19 UPDATING AND LIVING MAPS

Since maps typically have a broad scope they will date quickly as new studies appear. Annual updating is recommended, compared to every three years for a systematic review. Using machine learning to support searching and screening, as is possible in EPPI Reviewer, facilitates annual updates and the maintenance of living maps.

## Footnotes

1 The term outcome is used here to mean any effect from the intervention, which is common usage in maps and reviews. It is not the same usage as in the monitoring and evaluation literature which distinguishes outputs, outcomes, impact so that outcomes refer to high‐level indicators.

2 For this to be the cases, the map needs to have been sufficiently systematic. Where it has not been based on all components of a systematic search, for example, reference snowballing, then the authors of a map‐based review need carry out these additional components of the search strategy.

3 PICOS usually stands for Population, Interventions, Comparison, Outcomes and Study Designs which applies to effectiveness maps and reviews. Variations of the PICOS are used for other research questions. For example, in a qualitative map or review of intervention issues it may stand for Population, Issues, COntexts, and Study Designs.

4 INSPIRE stands for **I**mplementation and enforcement of laws, **N**orms and Values, **S**afe environment, **P**arent and Caregiver support, **I**ncome and economic strengthening, **R**esponse and support services, **E**ducation and life skills.

5 Both of these examples are illustrative, not recommendations.

6 The framework shown in Annex 2 also includes a definition of each label, and an example of a study coded with that label from the map. These other features are discussed below.

7 PRISMA stands for the Preferred Reporting Items for Systematic Reviews and Meta‐Analysis. See http://www.prisma-statement.org/.

8 This raises a seemingly esoteric point but an important one on which a decision needs to be made to ensure consistency in inclusion decisions. The inclusion criteria for primary studies and reviews will include a date, for example, 2000. The question to be decided is whether the screening of included reviews and maps uses that cut‐off date when screening the included studies in those reviews and maps. That is, if the inclusion cut‐off date is 2000 and a review published in 2009 includes one eligible study which was published in 1998, is that review included in the map?

9
http://www.casp-uk.net/casp-tools-checklists.

10
http://www.cardiff.ac.uk/specialist-unit-for-review-evidence/resources/critical-appraisal-checklists.

11
https://onlinelibrary.wiley.com/page/journal/18911803/homepage/submit-a-proposal.

12 See https://www.3ieimpact.org/evidence-hub/evidence-gap-maps/open-access-egm for further details.

13 The database component of the EGM software is under development at the time of writing this guidance.

## ANNEX 1 NOTES ON STAKEHOLDER CONSULTATION SESSIONS

The purpose of the stakeholder consultation is to either develop or validate the map framework, usually the intervention and outcome categories.

Both sessions described below need to begin with an orientation for the participants, which preferably includes presentation of an existing map or maps in related areas, allowing 5–10 min for the participants to look at the map. The scope of the proposed map needs to be explained, possibly with the commissioner explaining planned use.

### A1 Developing the framework

This approach requires small cards (preferably of two colours, say red for interventions and blue for outcomes), and tape or tack to fix the cards to the wall or a very large board. Prior to the session a set of cards should be prepared with an intervention or outcome written on each card—one intervention per intervention card and one outcome per outcome card. Prepare around 40 cards, including some which overlap or are duplicates.

After the introductory orientation, participants are handed blank cards and asked to write possible interventions and outcomes for the map. Stress one intervention, or one outcome, per card, as participants often list several on one card. Allow 5 min for this. As a variation, after the individual exercise, have participants work in small groups of 3–4 for five more minutes to collectively generate more cards.

All the intervention cards (red) are now piled together. The participants are asked to group these in piles of similar interventions. They can discard duplicates as they go. This works best with 6–10 people working around a large table on which the cards can be laid out. After grouping they can see if any of the cards in a group can be combined, with a new label covering all the combined cards. Each group needs a name, which is category label, with the individual cards as subcategories. Once completed, put the cards up on the wall as the map row headings.

Repeat the exercise for outcomes as the column headings.

If time allows try to fit some studies into the map as in the validation exercise described below.

### A2 Validating the framework

The rationale for the approach described here is that actively engaging participants in the map makes them more likely to critically reflect on it.

After the introductory orientation show some sample studies and screen them against the eligibility criteria. This is easiest done on title and abstract (T&A) only. First present one or two studies, showing the T&A, explicitly identifying the PICOS element which determine eligibility. Participants are then asked to screen 3–5 studies. It is best to have provided the T&As and a screening tool in the handout. Include at least one ineligible study. The studies you include may be selected to prompt discussion of specific issues, for example, including old studies or, most usually, not including before versus after study designs.

The same is now done for coding. That is, present a coding of a study (using the abstract, while emphasising in practice do not code on abstract) for intervention and outcomes. Then have the participants code the studies they screened as eligible. Again, the coding form should be in the handout. The feedback from this coding will prompt comments on what goes where.

Finally ask each participant to think of an intervention they work with or are familiar with and to fit that in the coding framework.

Small group work is generally more productive than plenary discussions. So, prior to plenary discussion ask groups of four to six people to discuss the framework and suggest possible modifications.

Finally, an open discussion on the framework should now take place so that suggestions from specific groups can be aired more generally.

## ANNEX 2 CODING FRAMEWORK WITH DEFINITIONS

**Table:**

1	Study design	Definitions	Example	
1.1	Systematic review	Review of primary studies adopting systematic approach, which includes: (a) search of at least three databases, (b) screening with explicit inclusion criteria, (c) coding and reporting of all relevant findings	Adams‐Guppy (2015). “A systematic review was conducted of research published utilising the MEDLINE, EMBASE, PsycInfo, CINAHL and SocIndex databases from inception to March 2015. A meta‐analysis was performed on studies that met the inclusion criteria, to determine if there were any significant pre‐ and postintervention effects on alcohol‐use”	
1.2	RCT	Random assignment of the intervention, including natural experiments	Essock (2006). “A total of 198 clients in two urban sites who had co‐occurring disorders and were homeless or unstably housed were randomly assigned to one of two treatment conditions and were followed for three years”	
1.3	Non experimental design with comparison group	Nonexperimental studies with comparison group including regression‐bassed designs (DD, RDD, IV, Matching, cohort, etc.)	O'Toole (2018). “We conducted a prospective, multicenter, quasi‐experimental, single‐blinded study at 2 VHA medical centers to assess health services use, cost, and satisfaction during 12 months among 2 groups of homeless veterans: (a) veterans receiving VHA homeless‐tailored primary care (Homeless‐Patient Aligned Care Team [H‐PACT]) and (b) veterans receiving traditional primary care services (PACT)”	
1.4	Before versus after design	Pre‐post outcome measurement with no comparison group	Cooley (2019). “Informed by personal construct theory, the present study explored the experiences of homeless young people living in sheltered accommodation (N = 116), when using strengths profiling at the start and end of a 10‐week, strengths‐based intervention”	
**2**	**Publication status**			
2.1	Completed	Completed: study findings are available in a published report or paper (published does not mean in a journal but in any form of a complete paper, NOT conference abstract or ppt)		
2.2	Ongoing	The research is still in progress. There is no published paper or report of study fundings	Parkes (2019). “This study will involve peer 'Navigators' providing practical and emotional support to homeless people who have substance use problems”	
**3**	**Study region**		The global region in which the study took place. For systematic reviews code against included studies. South America and Africa are not included as the map is restricyed to high income countries	
3.1	East Asia and Pacific		Note this includes Australia and New Zealand	
3.2	Europe and Central Asia			
3.3	North America			
**4**	**National Region: state in the US, or country in UK such England**			
4.1	Country			
		Australia		
		Canada		
		Czech Republic		
		Denmark		
		France		
		Ireland		
		Japan		
		Netherlands		
		Spain		
		South Korea		
		United Kingdom (UK)		
		United States of America (USA)		
4.2	City	Where available the city, town or county should be coded to aid with the geographical map produced by CHI		
**5**	**Population**	Population is not expected to be coded for all studies. It is used where the study is predominately about the named sub population		
5.1	Young People	Studies where the principle population group being studied are young people less than 18 years	Lynn (2014). “Improving youth mental health through family based prevention in family homeless shelters” “This exploratory study examines changes in suicidal ideation among a sample (N = 28) of homeless youth, ages 11–14, residing within family shelters in a large metropolitan area”	
5.2	Families with Children	Studies where the principle population group being studied are families with children	Edie (2018). “The purpose of this study is to help us learn what are the best services to promote young children's healthy development for families who are experiencing homelessness”	
5.3	Women and Girls	Studies where the principle population group being studied are women/girls	Lako (2018). This study examine the effectiveness of critical time intervention (CTI)—an evidence‐based intervention—for abused women transitioning from women's shelters to community living	
5.4	Elderly	Studies where the principle population group being studied are elderly (i.e., aged 65+)		
5.5	Discharge from Health Facilities	Studies where the principle population group being studied were in the process of being discharged from health facilities	Donkoh (2006). This review assesses the effectiveness of independent living programmes for young people leaving the care system	
5.6	People with/History of Mental Illness	Studies where the principle population group being studied are people with (or a history of) mental illness	Aquin (2017). This study attempted to determine if Housing First (HF) decreased suicidal ideation and attempts compared to treatment as usual (TAU) among homeless persons with mental disorders, a population with a demonstrably high risk of suicidal behaviour	
5.7	People with Complex Needs/Dual Diagnosis	Studies where the principle population group being studied are people with complex needs/dual diagnosis	Milby (2000). This study measured effectiveness of behavioral day treatment plus abstinence contingent housing and work therapy (DT+) vs behavioral day treatment alone (DT)… Participants (*N* = 110) met criteria for cocaine abuse or dependence, nonpsychotic mental disorders, and homelessness	
5.8	Veterans/Ex‐services	Studies where the principle population group being studied are veterans/ex‐service personnel (i.e., people who have left the Armed Forces)	Kasprow (2007). This study evaluated a modification of the critical time intervention (CTI) community case management model for homeless veterans with mental illness who were leaving Department of Veterans Affairs (VA) inpatient care	
5.9	People with Alcohol/Drug Issues	Studies where the principle population group being studied are people with alcohol/drug issues	Collins (2019). Randomized controlled trial of harm reduction treatment for alcohol (HaRT‐A) for people experiencing homelessness and alcohol use disorder	
5.1	LGBT community	Studies where the principle population group being studied identify as LGBT	Nyamathi (2016). Impact of Community‐Based Programs on Incarceration Outcomes Among Gay and Bisexual Stimulant‐Using Homeless Adults… This study was part of a randomized controlled trial designed to improve hepatitis knowledge and health promoting behaviors and subsequently decrease stimulant use and incarceration with 422 (G/B) homeless men between 18 and 46 years of age	
5.11	Survivors of Domestic Violence/Abuse	Studies where the principle population group being studied are survivors of domestic violence/abuse	Lako (2013). The study aims to examine the effectiveness of critical time intervention (CTI) for abused women and homeless people	
5.12	People with Disabilities	Studies where the principle population group being studied are people with disabilities	Bell (2015). This study evaluated the outcomes of a registered nurse‐led care management intervention for disabled Medicaid beneficiaries with high health care costs	
5.13	Migrants	Studies where the principle population group being studied are migrants	Chambers (2018). Housing‐vulnerable people included (but were not limited to) those who were homeless or had a history of homelessness, people with a history of mental illness, people with a learning disability, refugees and recent immigrants, young people leaving care and ex‐prisoners	
5.14	Ex‐prisoners	Studies where the principle population group being studied are ex‐prisoners (i.e., people who have previously spent time in a prison)	Jarrett (2012). This study aimed to see whether a Critical Time Intervention (CTI) in the first weeks post‐release effectively connects mentally ill prisoners with social, clinical, housing, and welfare services on leaving prison	
5.15	HIV Patients	Studies where the principle population group being studied have been diagnosed with HIV	Buchanan (2009). This study assesses the health impact of a housing and case management program, the Chicago Housing for Health Partnership, for homeless people with HIV	
5.16	Indigenous people	Studies where the principle population group being studied are ethnic groups who are the original inhabitants of a given region (first peoples, aboriginal peoples or native peoples)	No studies in map	
5.17	Leaving social care	Studies where the principal population group being studied are people leaving the social care system (in contact with social workers?)	Rashid (2004). This study assesses the outcomes of former foster care youth using transitional living programs in North Carolina	
**6**	**Intervention**	**Intervention subcategory**	**Definition**	**Example**
6.1	Legislation		Marked if any subcategory in this category is marked	
		Housing/homelessness legislation	Legislation pertaining to availability of/access to housing, or the rights of those experiencing homelessness	None in map
		Welfare benefits	Legislation for welfare programmes to help people experiencing homelessness, or to help prevent people who are at risk of becoming homeless from losing their home	Riccio (2019). “The U.S. Department of Housing and Urban Development (HUD) launched the Rent Reform Demonstration to test important modifications to the federal government's traditional rent policy to determine whether the changes can improve tenants' success in the labor market and reduce the administrative burden on PHAs in operating the voucher program”
		Health and social care	Legislation for access to health and social care to help people experiencing homelessness, or to help people who are at risk of becoming homeless	Zewede (2019). “Objectives: To evaluate the effect of the Affordable Care Act (ACA) Medicaid expansions on national rates of home eviction and eviction initiation in the United States”
6.2	Prevention		Marked if any subcategory in this category is marked	
		Welfare and housing support	State contribution towards housing costs and other welfare payments and services, whether directly made to tenants or indirectly paid to service provider (e.g., landlords—examples in the UK: Local Housing Alliance, Universal Credit, etc.; US: vouchers) from the state or nonstate actors. This includes other welfare benefits such as childcare if studied in the context of homelessness	Wolitski (2010). “Homeless/unstably housed people living with HIV/AIDS (N = 630) were randomly assigned to immediate Housing Opportunities for People with AIDS (HOPWA) rental assistance or customary care”
		Housing supply	Policies promoting the development of new housing supply that is affordable and accesible (whether for social or private purposes)—this includes the construction, conversion of homes, and repurposing. Interventions comprise changes to legisltation, financing mechanisms and other support for developers and those conditioning units for these purposes	None in map
		Mediation and conciliation	Counselling and mediation of conflicts, usually between young people and their family so they may avoid becoming homeless or reduce other risky behaviours (Landlord‐tenant mediation is a separate category)	Millburn (2012). “a family‐based program can also result in significant reductions in risky behavior… We aimed to re‐engage youth with their families”
		Landlord‐tenant mediation	Mediation between landlords and tenants to encourage landlords to accept tenants with history of homelessness, substance abuse, and so forth, and to address conflicts. This may include, but is not limited to mediation around arrears, noise and substance abuse, damage to property, eviction, and so forth	Wade (2009). “The Advocacy & Benefits Program offers alternative homeless prevention and intervention services such as housing services (shelter referrals, housing search, rental and mortgage arrearage, help, first, last & security deposits, and utilities help), legal assistance (landlord tenant negotiation, eviction issues, court representation)….”
		Discharge interventions	Provision of services, including accommodation, to people being discharged from institutions (care, hospitals, prison, armed forces) to avoid people being discharged into homelessness. This may include coordination between agencies, accomodation, and other services tailored to their needs. It refers to both interventions while in the insitution and community‐based interventions focused on recently discharged persons	Tomita and Herman (2012). “This study examined the impact of critical time intervention (CTI) in reducing rehospitalization among formerly homeless individuals with severe and persistent mental illness after discharge from inpatient psychiatric treatment”
6.3	Services and outreach		Marked if any subcategory in this category is marked	
		Soup runs	Provision of food in street settings to people experiencing homelessness	Sharea et al. (2018). “This review aimed to assess effectiveness of interventions designed to prevent or treat malnutrition in homeless problem‐drinkers… Nine studies evaluated educational and support interventions, five food provision, and three supplement provision”
		In‐kind support	Provision of clothing, hygiene products, household items, and so forth	O'Toole (2015). “Clinic Orientation Arm… additional resources available at the clinic (clothes, hygiene kits, food, and benefits representatives, available to all homeless Veterans regardless of primary care enrollment)…”
		Day centres	Centres open only during the day to provide food and services for people experiencing homelessness. This code is used if the day centre itself is being evaluated in the study rather than being the setting for the intervention	Bond (1990). “Outcomes were examined after 1 year for 82 clients, averaging over 17 lifetime psychiatric hospitalizations, randomly assigned either to ACT or to a drop‐in (DI) center”
		Outreach	Outreach refers to work with people sleeping rough or in temporary or unstable accommodation. Outreach workers go out, including late at night and in the early hours of the morning, to locate people who are rough sleeping or work with day centres, shelters, and so forth. The role of outreach teams varies but usually outreach workers seek to engage with people and check their immediate health and wellbeing, collect basic information about their situation, facilitate access to emergency accommodation or other accommodation (such as hostels or Housing First), and inform them about day centres and other services they might have available. Outreach models vary and may include enforcement (e.g., police officials) to remove people from the streets or enforce specific behaviours	Stergiopoulous (2010). “This article describes a collaborative interagency multidisciplinary outreach team”
		Reconnection of rough sleepers	Reconnecting people experiencing homelessness (rough sleepers) or at risk of homelessness (e.g., dischargees) to their “home” location (usually another city, state or country where they have networks, access to services, etc.) by providing the cost of transport for relocation	No studies in the map
		Psychologically informed interventions for service deliverers	Interventions informed by a psychological theory or approach, for example, motivational interviewing and mindfulness. It includes interventions aimed at service deliverers that help them consider the psychological profile of those living with homelessness. Examples of features of a PIE are psychological awareness, staff training and support, and a learning approach	Bender (2015). “Using a randomized experimental design, the current study pilot tests an intensive (3 day), skill‐building intervention to train homeless youth (N = 97, ages 18–21 years) to practice mindfulness and avoid risks”
		Case management (inc. Critical Time Intervention)	Individual‐level approach to ensure coordination of services. The case worker (can be social worker or dedicated case worker from another agency) works directly with the client to ensure that the client has access to all applicable services, for example, health, training and social activities. A specific application of the case work approach is critical time intervention (CTI) which provides a person (or family) in transition between types of accommodation and at risk of homelessness with a period of intensive support from a caseworker. The caseworker will have established a relationship with the client before the transition—for example, before discharge from hospital or prison.Critical time intervention involves three stages: (a) direct support to the client and assessing what resources exist to support them, (b) trying out and adjusting the systems of support as necessary, and (c) completing the transfer of care to existing community resources	Klinkenberg (1998). “This study examined the role of the helping alliance in case management with homeless persons who have a severe mental illness”
		Service coodination, colocation or embedded in mainstream services	System‐based approaches to ensuring coordination of service delivery. Coordination may refer to ensuring communication between relevant services. Coordination also includes providing services in the same location or adjacent to mainstream services. Colocation refers to multiple services being available in the same physical location (e.g., housing and job search services in the same location). Embedded refers to services being integrated in the same place (e.g., housing and other services within a hospital context). A specific example is coordinated assessment. Refers to case workers making broad assessments of people at risk as homelessness on different factors that affect their risk. Try to ensure different services employ the same assessment tools to standardise practice	Karper (2008). “This study, without creating new services, worked within the existing structure to create a new process to improve outcomes for a high risk group for whom effective access to services and compliance with treatment has been lacking. The decision to utilize care coordination and improve the collaboration between a hospital and homeless shelter allowed for the validation of the program. Institutional goals of this collaboration were to improve communication and trust between the two institutions and to develop and test a process for the coordination of care for the homeless. The objective of this study was to test the hypothesis that improving the coordination of care of homeless people with substanceuse and psychiatric disorders will result in a significant improvement in psychiatric symptoms without increasing utilization of residential services”
		Veterinary services	Access to veterinary services for pets of people experiencing homelessness	None in map
		Legal advice	Legal assistance and advice delivered away from primary service/office to the homeless population	Vaclavik (2018). “HPRP offered a menu of housing and financial services that were administered based on family need. Housing services included assistance finding affordable housing, legal services, and housing stabilization services. Financial services included rental assistance, security deposits, utility deposits, moving costs, and rental or utility arrears”
6.4	Accommodation based services	Shelters	Homeless shelters are a basic form of temporary accommodation where a bed is provided in a shared space overnight. One of the key features of a homeless shelter is that it is transitional and an option for those homeless who are not yet eligible for more stable accommodation. Shelters are not usually seen as stable forms of accommodation as the individual must vacate the space during daytime hours with their belongings. One of the key differences with hostels is the need to vacate the premises during the day	Clark (2018) “Objectives. To describe longitudinal health service utilization and expenditures For homeless family members before and after entering an emergency shelter”
		Hostels	Hostels for homeless people are designed provide short‐term accommodation, usually for up to two years depending on available move‐on accommodation. Typically shared accommodation projects with individual rooms and shared facilities including bathrooms and kitchens. Hostels have staff on site 24 hr a day and during the daytime provide support to residents on issues including welfare benefits and planning their move from the hostel into more medium to long‐term accommodation	Parkes (2019). “Phase 1 (months 1–3) will address objectives 1 and 2: 1. Develop an intervention using co‐production methods for use in community outreach/hostel settings”
		Temporary accommodation	Temporary accommodation includes a range of housing options, which may include hostels and shelters. The key characteristic is that TA is NOT considered an emergency solution and is embedded in the legal framework	Rashid (2004). “The goals of this study were to (a) assess the outcomes of former foster care youth using transitional living programs and (b) compare outcomes achieved by former foster care youth who participated in an employment training program with similar youth who did not”
		Host homes	Emergency Host homes are emergency short‐term placements in volunteers’ own homes in the community for people who are homeless or at risk of homelessness. Hosting services are often aimed at young people with low support needs, but exist for other groups too, such as people who have been refused asylum	No studies in map
		Rapid Rehousing	Rapid rehousing places those who experiencing homelessness into accommodation as soon as possible. The intervention provides assistance in finding accommodation, and limited duration case work to connect the client to other services	Hunter (2020). “Breaking Barriers: A Rapid Rehousing and Employment Pilot Program for Adults on Probation in Los Angeles County”
		Housing first	Housing First offers accommodation to homeless people with multiple and complex needs with minimal obligations or conditions being placed upon the participant. Housing First provides safe and stable housing to all individuals, regardless of criminal background, mental instability, substance abuse, or income	Poremski (2016). “Effects of Housing First on Employment and Income of Homeless Individuals: results of a Randomized Trial”
		Social housing (with or without support)	Housing that is provided in the social sector. It may sometimes be provided alongside support services, this may be temporary or permanent. Examples of support that may be provided are health and money management (excluding Housing First and Rapid Rehousing). This is based on an institutional setting	Tabola (2010). “Supported housing is a service model that couples provision of independent housing with provision of community‐based supports for individuals with psychiatric disabilities at risk of homelessness”
		Private rental sector (with and without support)	Housing that is provided in the private rental market where the tenant is fully responsible. This may or may not include additional support services as the focus is on the type of tenancy agreement (private)	Tsai (20110). “we used an observational design to compare four groups: (a) obtained independent housing without a voucher; (b) obtained independent housing with a voucher; (c) housed in another individual's place; or (d) were not yet housed (i.e., living in an institution, hotel, or are homeless situation)”
		Continuum of care	An approach to accommodation whereby people experiencing homelessness move through different forms of transitional accommodation until they are deemed “housing ready” (e.g., stopped substance abuse) and allocated independent settled housing	Greenwood (2005). “control participants received contact information for two social service providers that followed the Continuum of Care model”
6.5	Employment	Mentoring, coaching and in‐work support	Mentoring and coaching to support job search including activities like practice interviews, review CVs, and so forth, and on the job support for work performance	Bartle‐Haring (2012). “The mentor provided advice and encouragement and discussed strategies for solving problems related to the adolescent's living situation, finances, staying sober, job finding, obtaining bank accounts, and making new friends”
		Flexible employment	Employment which can accommodate needs for the person experiencing homelessness	None in map
		Vocational training and unpaid work experiences	Unpaid job placement or vocational training to provide work experience for people experiencing, or at risk of, homelessness	Milby (2010). “One group (n = 103) received abstinence‐contingent housing, vocational training, and work; another group (n = 103) received the same intervention plus cognitive behavioral day treatment”
		Paid work experiences	Paid job placement to provide work experience for people experiencing, or at risk of, homelessness	Koffamus (2011). “Participants (n = 124) were randomly assigned to conditions either requiring abstinence from alcohol to engage in paid job skills training (Contingent Paid Training group), offering paid job skills training with no abstinence contingencies (Paid Training group) or offering unpaid job skill training with no abstinence contingencies (Unpaid Training group)”
6.6	Health and social care	Health services (physical and mental)	Providing direct access to, or facilitating access to, physical and mental health services for people experiencing homelessness	Nyamathi (2006). “To compare the effectiveness of an intervention program employing nurse case management and incentives (NCMI) vs. a control program with standard care and incentives on completion of LTBI treatment; and 2) to compare the impact of the two programs on tuberculosis (TB) knowledge among participants”
		End of life care	End of life care for people experiencing or at risk of homelessness	Song (2010). “OBJECTIVE: To determine whether homeless persons will complete a counseling session on advance care planning and fill out a legal advance directive designed to assess care preferences and preserve the dignity of marginalized persons”
		Addiction support	Services for people experiencing, or at risk of, homelessness who have substance misuse problems (including alcohol and other substances)	Kertesz (2007). “This study examined the percentage of cocaine‐using homeless persons (all with psychiatric distress) attaining stable housing and employment 12 months after entering a randomized trial of intensive behavioral day treatment, plus one of the following for 6 months: no housing; housing contingent on drug abstinence; housing not contingent on abstinence”
6.7	Education and skills	Life and social skills training	Life and social skill training including socio‐emotional skills, financial literacy (money management), tenancy management, and how to deal with ones home; for people, experiencing or at risk of homelessness	Medalia (2017). “Ninety‐one homeless youth were randomized to receive either targeted cognitive training (cognitive remediation) or general cognitive activation (computer skills training)”
		Mainstream education	General education (e.g., Maths, Literacy, IT) for people experiencing, or at risk of, homelessness	Sacks (2004). “The primary aim of this study is to evaluate the impact of a group exercise intervention on activity levels in people who are homeless or at risk of homelessness in central London, UK”
		Homelessness‐specific programmes in schools	Homelessness‐specific programmes which take place in an educational setting (i.e., schools)	Kadoura (2015). “A pilot study was conducted with the purpose of developing, implementing, and evaluating the effectiveness of the Brighter Futures for Preschoolers Program (BFPP) for high‐risk homeless families of preschoolers”
		Recreational and creative activities	Recreational, social (e.g., social clubs) and creative (e.g., theatre) activities for people experiencing homelessness	Stringer (2019) “The primary aim of this study is to evaluate the impact of a group exercise intervention on activity levels in people who are homeless or at risk of homelessness in central London, UK”
6.8	Communication	Advocacy campaigns	Campaigns by 3rd sector organisations which aim to improve awareness of the general public of homelessness, its causes, and its solutions, and promote rights of the homeless	No studies in the map
		Public information campaigns	Campaigns by government organisations which aim to improve awareness of the general public of homelessness, its causes, and its solutions, and promote rights of the homeless	No studies in the map
		Service availability	General communication activities to raise awareness among people experiencing homelessness, or at risk of homelessness, of the services available to them. Does not include case management, discharge, and so forth, which provides information or connects individuals to services	No studies in the map
6.9	Financing	Social impact bonds	Performance‐based financing for organisations commissioned to provide services to people experiencing homelessness. Not these are not interventions in themselves, but payment mechanisms for service deliverers	Spurling (2017). “The London Homelessness Social Impact Bond was a four year programme designed to bring in new means of financing interventions, and encourage innovative approaches, to address rough sleeping among an entrenched group of rough sleepers in London”
		Direct financial support from public	Money given directly by individuals to those experiencing or at risk of homelessness	No studies in the map
**7**	**Outcomes**	**Outcome subcategory**	**Definitions**	
7.1.	Capabilities and Wellbeing	Social connectedness and social networks (including loneliness)	Community engagement and social connectedness, for example, social networks and loneliness	Patterson (2014). This study examines community engagement among homeless adults with mental illness 6 and 12 months after random assignment to Housing First (independent apartments or congregate residence) with support services or to treatment as usual (TAU)
7.1.1		Improved skill and self care	Improved skill and self care including all life skills	Helfrich (2011). This study investigated the effectiveness of a situated learning theory, a life skills intervention on improved and retained life skills knowledge
7.1.2		Overall wellbeing and quality of life	Overall wellbeing or quality of life including happinness	Aubry (2015) This a parallel group RCT examined the effectiveness of housing first on housing stability and quality of life after one year of enrolment
7.2.1	Cost	Cost (TOTAL)	Cost related outcomes/indicators. This includes cost effectiveness, cost per participant and saving	
7.2.2		Cost effectiveness	Cost effectiveness as cost per outcome in absolute or relative terms	Wolff (1997). This study compared the cost‐effectiveness of three approaches to case management for individuals with severe mental illness who were at risk for homelessness: assertive community treatment alone, assertive community treatment with community workers, and brokered case management
7.3.3		Cost per participant	Cost per participant	O'Toole (2018). This study was a prospective quasi‐experimental trial that assessed the impact of enrolment in 2 groups of homeless veterans: (a) veterans receiving VHA homeless‐tailored primary care (Homeless‐Patient Aligned Care Team [H‐PACT]) and (b) veterans receiving traditional primary care services (PACT). Annual costs per patient were found to be significantly higher in the PACT group than the H‐PACT group
7.3		Saving	Cost savings from interventions (e.g., “this policy would reduce the number of ambulance/police incidents and save the government money”)	Pauly (2018). The findings of this study suggests that having an MH Clinical Pharmacy Specialist clinic in the HPACT clinic is associated with cost savings from a preventative‐care standpoint
7.3.1	Crime and justice	Crime and justice (TOTAL)	Crime and justice outcomes/indicators. This includes arrest and imprisonment, recindivism and victims of crime	
7.3.2		Offending	Any measure or record of any recognized crime (violent/nonviolent/any other offence)	LeClair (2019). “Two studies from a randomised controlled trial found no effect of Housing First on arrests compared to treatment as usual”
7.4		Arrest and imprisonment	Crime perpetrator outcomes/indicators such as arrest, conviction and imprisonment	Frisman (2009). This study reports that the participants assigned to Assertive Community Treatment (ACT) group shows a significant greater reduction in alcohol use and were less likely to go to jail than those in standard clinical case management
7.4.1		Recidivism	Tendency of a convicted criminal to reoffend	Somers (2013). This study confirms that HF programs – particularly those using the scattered site format ‐ promote reductions in offending and reconviction among people who were previously homeless and have a current mental disorder
		Victims of crime	Outcomes/indicators about those experiencing and at risk of homelessness being victims of crime	Jonker (2015). This review concludes that interventions provided during and after women's stays in shelters are effective in improving mental health outcomes, in reducing the incidence of re‐abuse and in improving social outcomes
7.4.2	Employment and income	Employment and income (TOTAL)	Employment and income outcomes/indicators. This includes access to welfare benefits, earned income, employment status, forced labour and sex work	
7.4.3		Access to welfare benefits	Access to welfare benefits as outcomes/indicators	Grace (2014). This study reports that the YP intervention to progress along a pathway that will lead to more sustainable employment and housing outcomes. This was assessed by examining housing stability, suitability and affordability; and income from both employment and Centrelink
7.5		Earned income	Earned income (e.g., salary or wages)	Riccio (2019). The results indicate that when the findings for all four PHAs are combined, the new policy did not generate statistically significant increases in tenants’ average earnings over the first 18 months of followup
7.5.1		Employment status	Employment status (e.g., employed full time, self employed, unemployed, etc.)	Poremski (2016). This study concluded that the ICM recipients had lower odds of obtaining employment compared with the control group with moderate needs. The odds of obtaining employment among ICM recipients increased but their employment rate never exceeded that of the control group
7.5.2		Forced labour and sex work	Forced labour and sex work (e.g., slavery or prostitution)	No studies in the map
7.5.3	Health	Health (TOTAL)	Health outcomes/indicators. This includes abstinence from substance abuse, access to mainstream health care, harm reduction, mental health status and physical health and nutrition status	
7.6		Substance abuse	Abstinence from substance abuse including both alcohol and tobacco (e.g., 12 months without alcohol or drugs)	Kashner (2002). This study reported that the participants addigned for work therapy were likely to initiate outpatient addiction treatments and experience fewer drug and alcohol problem
7.6.1		Access to mainstream health care	Access to and utlization of mainstream health care as outcomes/indicators (e.g., registered with a local general practice doctor)	Wolitski (2010). This study demonstrated PLHIV assigned to HOPWA groups had significant reductions in medical care utilization and improvements in self‐reported physical and mental health
7.6.3		Mental health status	Mental health status (e.g., diagnosed with conditions such as depression, anxiety, psychosis, personality disorder, etc.)	Lynn (2014). The finding of this study indicated that health education programs integrating a family strengthening approach hold promise for positively impacting mental health outcomes for vulnerable youth
7.7		Physical health and nutrition status	Physical health or nutrition (e.g., life expectancy, dietary intake, anthropometric indicators)	Randers (2012). This finding of this pre‐post study suggested that the high exercise intensity during street soccer and regular street soccer training can be used as an effective activity to promote physical fitness and cardiovascular health status for homeless men
		Risky behaviour	risky behavior as outcomes/indicators (eg. early onset of sexual activity or unsafe sexual practices, risky driving, antisocial behavior etc.)	Rew (2017). This study demonstarted that particiants asigned to a brief intervention for Homeless Female Youths had greater self‐confidence in negotiating safer sex practices than comparison participants
7.7.1	Housing Stability	Housing stability (TOTAL)	Housing stability outcomes/indicators. This includes accommodation status and satisfaction with housing	
7.7.2		Accommodation status	Accommodation status or quality of housing as outcomes/indicators (e.g., living independently, living in temporary accommodation, sleeping on the streets)	Benston (2015). This systemic review found that a majority of participants placed in experimental housing programs with case management support remained in housing for at least one year or experienced more days housed than homeless relative to a comparison group
7.7.3		Satisfaction with housing	Satisfaction with housing (subjective, objecitve measures are in accommodation status)	Schutt (1997). This study reports that the subjects were more satisfied with their residential accommodations after moving into permanent housing and liked independent housing more than group living
7.7.4	Public attitudes and engagement	Public attitudes and engagement (TOTAL)	Public attitudes and engagement. This includes fundraising, public understanding, support for intervention, and engagement in homelessness related activities	
7.8		Fundraising	Charity fundraising	No studies coded
7.8.1		Public understanding	Public understanding as outcomes/indicators (e.g., hostility or empathy towards homeless people)	No studies coded
7.8.2		Support for intervention	Level of support for the intervention as outcomes/indicators by beneficiaries, other stakeholders and the public	Beggs (2016). All participants indicated they would attend a future class led by pharmacy students
7.8.3		Engagement in homelessness related activities	Public engagement in homeless related activities as outcomes/indicators (e.g., number of volunteer applicants)	

### Consultation process for this document

The initial draft was produced the Campbell EGM Working Group in January to March 2018. This working group (who are the named authors) were also sent methodological queries when the guidance was being further developed in summer 2019.

A revised version was subject to the following consultation process:

Stage 0—internal (Campbell secretariat staff) (February 2020)
Stage 1—the working group (March 2020)
Stage 2—knowledgeable friends, that is, producers and commissioners of maps (April to May 2020)
Stage 3—CGs and public (June to July 2020)

Submission of guidance for publication August 2020
